# Ginsenoside Rb1 protects against ischemia/reperfusion-induced myocardial injury via energy metabolism regulation mediated by RhoA signaling pathway

**DOI:** 10.1038/srep44579

**Published:** 2017-03-22

**Authors:** Yuan-Chen Cui, Chun-Shui Pan, Li Yan, Lin Li, Bai-He Hu, Xin Chang, Yu-Ying Liu, Jing-Yu Fan, Kai Sun, Quan -Li, Jing-Yan Han

**Affiliations:** 1 Department of Integration of Chinese and Western Medicine, School of Basic Medical Sciences, Peking University, Beijing 100191, China; 2 Tasly Microcirculation Research Center, Peking University Health Science Center, Beijing 100191, China; 3 Key Laboratory of Microcirculation, State Administration of Traditional Chinese Medicine of the People’s Republic of China, Beijing 100191, China; 4 Key Laboratory of Stasis and Phlegm, State Administration of Traditional Chinese Medicine of the People’s Republic of China, Beijing 100191, China; 5 Beijing Laboratory of Integrative Microangiopathy, Beijing 100191, China; 6 Department of Cardiology, Beijing China-Japan Friendship Hospital, Beijing 100029, China

## Abstract

Cardiac ischemia and reperfusion (I/R) injury remains a challenge for clinicians. Ginsenoside Rb1 (Rb1) has been reported to have the ability to attenuate I/R injury, but its effect on energy metabolism during cardiac I/R and the underlying mechanism remain unknown. In this study, we detected the effect of Rb1 on rat myocardial blood flow, myocardial infarct size, cardiac function, velocity of venule red blood cell, myocardial structure and apoptosis, energy metabolism and change in RhoA signaling pathway during cardiac I/R injury. In addition, the binding affinity of RhoA to Rb1 was detected using surface plasmon resonance (SPR). Results showed that Rb1 treatment at 5 mg/kg/h protected all the cardiac injuries induced by I/R, including damaged myocardial structure, decrease in myocardial blood flow, impaired heart function and microcirculation, cardiomyocyte apoptosis, myocardial infarction and release of myocardial cTnI. Rb1 also inhibited the activation of RhoA signaling pathway and restored the production of ATP during cardiac I/R. Moreover, SPR assay showed that Rb1 was able to bind to RhoA in a dose-dependent manner. These results indicate that Rb1 may prevent I/R-induced cardiac injury by regulation of RhoA signaling pathway, and may serve as a potential regime to improve percutaneous coronary intervention outcome.

Myocardial infarction is an emergency condition with high incidence and mortality^1^. Thrombolytic therapy or primary percutaneous coronary intervention (PCI) is currently the most effective strategy to improve the clinical outcome for the patients presenting with acute myocardial infarction. PCI restores blood flow in myocardium, however, it frequently results in reperfusion injury^2^.

Ischemia and reperfusion (I/R) injury is a process that initiates in the ischemia phase due to hypoxia and is exacerbated in reperfusion phase, in which mitochondria, particularly the ATPase, plays a central role^3^. ATPase is a multisubunit complex consisting of 15 subunits including ATP 5D in mammals, which integrate into two domains, F1 and Fo. ATPase is reported to respond to ischemia and reperfusion by changing the expression of subunit protein (s)^4^,^5^ leading to ATPase dysfunction. ATPase dysfunction may impose dual deleterious impacts on the tissue underwent I/R, which, on one hand, results in ATP depletion thus affects all the energy consuming processes in the cell, such as cell contraction and ion pumps, as well as triggers the production of reactive oxidative species, and disrupts the mitochondrial membrane potential (ΔΨm), on the other hand, is implicated in the progressing of apoptosis^6^. Thus, maintenance of mitochondria structure and function is expected to be a promising strategy for protection of cardiac I/R injury.

Evidence has accumulated showing small GTPase and their regulators as important players of the cardiovascular physiology that control a large panel of cardiovascular functions^7^,^8^. Among them, signaling pathways of Rho family have been reported to exert a variety of effect on cellular structure and function^9^. Studies have shown that RhoA/Rho-associated coiled-coil containing protein kinase-1 (ROCK-1), a major downstream effector of RhoA, is involved in I/R injury and, particularly, has a regulatory effect on energy metabolism. RhoA/ROCK activation was found to aggravate heart I/R injury through mediating myocardial apoptosis, inflammation inducing cardiac remodeling and fibrosis^10^,^11^. However, the strategy directing RhoA to fight against I/R injury is so far limited.

Ginsenoside Rb1 (Rb1) is a major effective ingredient of *Panax ginseng* C. A. Mey, a traditional herbal medicine frequently used in eastern countries. Rb1 has been reported to protect against I/R injury. However, the underlying mechanisms are not fully understood. QiShenYiQi Pills^®^ (a compound Chinese medicine containing Rb1 as a major component) has proven able to regulate energy metabolism deficiency and expression of ATP 5D in cardiac I/R injury^12^,^13^. Our previous study reported that notoginsenoside R1 (NR1) ameliorated I/R induced myocardial injury by regulating levels of ROCK1 and ATP5D, implying a likely link between RhoA signaling pathway and energy metabolism regulation^4^. However, it is so far unclear whether Rb1 can improve I/R-induced cardiac energy deficiency and, if yes, what is the role of RhoA signaling pathway in the process.

In the present study we explored the effect of Rb1 on cardiac injury by I/R challenge, intending to gain more insight into the underlying mechanism, especially focusing on the potential involvement of RhoA signaling pathways in the regulation of energy metabolism and myocardial structure.

## Results

### Rb1 diminishes I/R-induced myocardial infarct size and tissue injury

**Figure 1:**
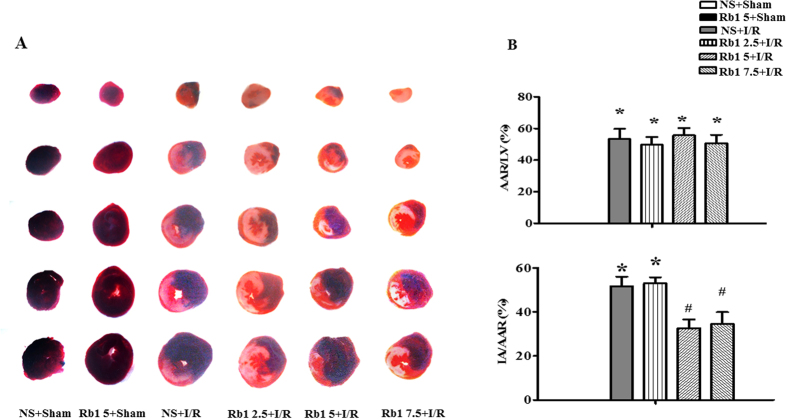
Effect of Rb1 on rat myocardial infarct size after I/R. (**A**) Representative slices of LV stained with Evans blue-TTC in different groups. The infarct zone was stained white, non-infarction zone was blue, and AAR was pink. (**B**) and (**C**) Quantification of AAR/LV and IA/AAR after I/R in various groups. Results are presented as mean ± S.E.M (n = 6). *p < 0.05 vs. sham group, ^#^p < 0.05 vs. I/R group.

Myocardial infarct was assessed by Evans blue-triphenyltetrazolium chloride (TTC) staining after 90 min of reperfusion. Displayed in Fig. 1A are the representative heart slices in different groups, in which the pink color denotes ischemic myocardial area, the white the infarction area and the blue normal area. Obviously, no infarct was noticed in myocardial tissue slices from sham groups. In contrast, myocardial tissue slices in I/R group exhibited apparent infarct. As compared to I/R group, heart slices from Rb1 treatment at 2.5, 5 and 7.5 mg/kg/h groups had similar area of ischemic region, while Rb1 5 and 7.5 mg/kg/h treatment groups had obviously smaller infarct area. This result is confirmed by quantification of area at risk/left ventricle (AAR/LV) and infarct area/area at risk (IA/AAR), as shown in Fig. 1B and C, respectively. AAR/LV increased obviously in I/R group compared with sham with no significant difference being found between I/R group and Rb1 treatment groups at the three doses tested. I/R challenge led to a significantly increased IA/AAR in comparison with sham groups, which was prevented significantly by treatment with Rb1 at 5 and 7.5 mg/kg/h, indicating the protective effect of Rb1 at the two doses on I/R-induced myocardium infarct, and we used 5 mg/kg/h in all the following experiments.

### Rb1 restores myocardial blood flow during I/R

**Figure 2:**
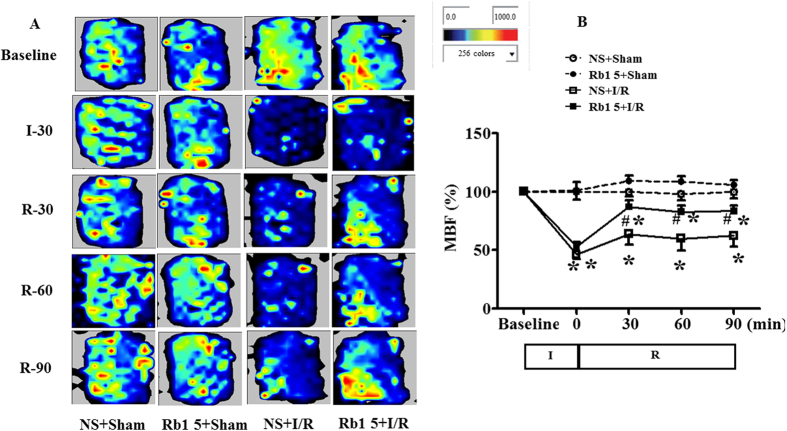
Effect of Rb1 on MBF during I/R. (**A**) MBF images of rats from different groups acquired by Laser Scanning Doppler Perfusion Imager at indicated time points. The different colors represent the magnitude of MBF, with blue to red defining low to high. (**B**) Change of MBF with time in rats from various groups. The linear mixed effect models were analyzed for repeated measurement data, and least squares means were calculated between the groups of different time points. Data are expressed as the means ± S.E.M (n = 6). *p < 0.05 vs. sham group, ^#^p < 0.05 vs. I/R group.

The myocardial blood flow (MBF) in different groups was assessed by the Laser Scanning Doppler at different time points, and the representative images are presented in Fig. 2A. MBF in NS + sham and Rb1 + sham groups kept nearly constant over the period of observation. In contrast, ischemia caused prominent decrease in MBF in I/R group, which persisted till the end of the observation. Rb1 prevented MBF from decrease by I/R significantly. The time courses of MBF changes in different groups are depicted in Fig. 2B, which verified the survey results from Fig. 2A. Of notice, MBF in I/R group decreased to nearly 40% of baseline immediately after ischemia without recovery over the observation. Rb1 at 5 mg/kg/h significantly restored the decrease in MBF at 30 min after reperfusion, this effect persisted until the end of observation.

### Rb1 ameliorates the impairment of heart function induced by I/R

**Figure 3:**
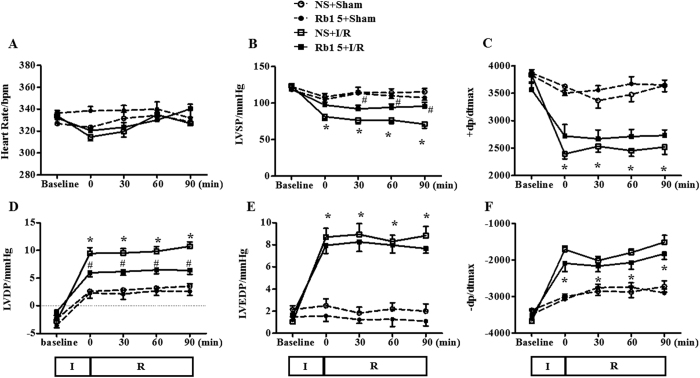
Effect of Rb1 treatment on rat heart function. Presented are the time courses of HR (**A**), LVSP (**B**), +dp/dtmax (**C**), LVDP (**D**), LVEDP (**E**) and −dp/dtmax (**F**) in different groups. The linear mixed effect models were analyzed for repeated measurement data, and least squares means were calculated between the groups of different time points. Data are expressed as the means ± S.E.M (n = 6). *p < 0.05 vs. sham group, ^#^p < 0.05 vs. I/R group.

Heart function of rats in different groups was determined, and the results are shown in Fig. 3A through F. As compared with sham groups, I/R elicited a significant decrease in left ventricular systolic pressure (LVSP) and left ventricular maximum upstroke velocity (+dp/dtmax), and an inclement in left ventricular developed pressure (LVDP), left ventricular end diastolic pressure (LVEDP) and left ventricular maximum descent velocity (-dp/dtmax), indicating an impairment of heart function. Obviously, Rb1 attenuated the impairment in LVSP and LVDP, while had no effect on disorder in other indexes.

### Rb1 ameliorates the decrease in red blood cell velocity in coronary venules induced by I/R

**Figure 4:**
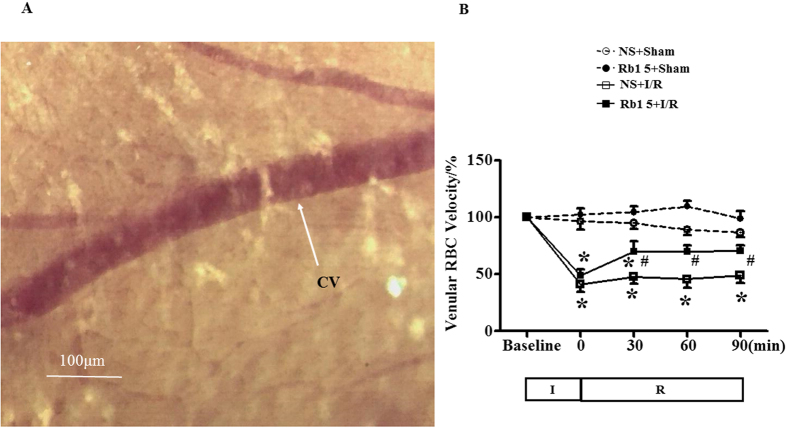
Effect of Rb1 on RBC velocity in coronary venule in rat exposed to I/R. (**A**) The cardiac coronary microcirculation. CV, coronary venule. Bar = 100 μm. (**B**) The time course of change in RBC’s velocity in coronary venules of different groups. Data are expressed as the means ± S.E.M (n = 6). *p < 0.05 vs. sham group, ^#^p < 0.05 vs. I/R group.

Red blood cells (RBCs) moving inside coronary venules were visible under microscope, which were recorded with a high-speed video camera (Fig. 4A). The stored images were replayed at 25 frames/s, and the RBC moving velocity in venules was evaluated. Figure 4B illustrates the change in RBC velocity with time in venules from different groups. The RBC velocity in venules in the sham groups remained nearly unchanged during the observation. By contrast, ischemia elicited a significantly decreased RBC velocity in I/R group, which remained at a relatively low level by 90 min of reperfusion. Treatment with Rb1 significantly restored the RBC velocity decrease induced by I/R starting from 30 min after reperfusion.

### Rb1 diminishes I/R-induced cardiomyocyte injury

**Figure 5:**
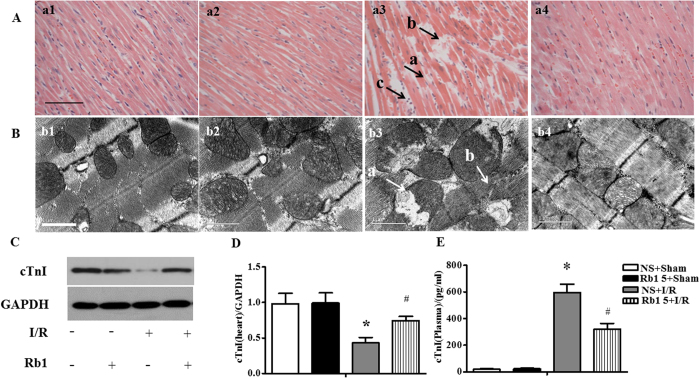
Effect of Rb1 on myocardial morphology after I/R. (**A**) Representative photographs of myocardium stained by HE in different groups. a1: NS + Sham group, a2: Rb1 5 + Sham group, a3: NS + I/R group, a4: Rb1 5 + I/R group. Bar = 50 μm. a: ruptured myocardial fiber, b: edema in between myocatdial fibers, c: recruited leukocytes. n = 3. (**B**) Presented are the representative electron micrographs of myocardium from different groups. b1: NS + Sham group, b2: Rb1 5 + Sham group, b3: NS + I/R group, b4: Rb1 5 + I/R group. Bar = 1 μm. a: swelling mitochondria, b: disrupted myofibril. n = 3. (**C** and **D)** The representative Western blot bands and semi-quantitative analysis of cTnI in in heart at 90 min after reperfusion following 30 min ischemia. The samples derived from the same experiment and gels were processed in parallel. Full-length gels are shown in Supplementary Fig. S5 with indication of molecular size. Data are expressed as the means ± S.E.M (n = 4). *p < 0.05 vs. sham group, ^#^p < 0.05 vs. I/R group. (**E**) cTnI level tested by ELISA in plasma after I/R. Data are expressed as the means ± S.E.M (n = 8) *p < 0.05 vs. sham group, ^#^p < 0.05 vs. I/R group.

The histology of myocardium stained by hematoxylin-eosin (HE) in different groups was examined with the results presenting in Fig. 5A. Compared with sham group, apparent alterations were visible in the surrounding infarction areas of myocardial tissues from I/R group, including interstitium edema, disruption of myocardial fibers and leukocyte recruitment. However, Rb1 treatment remarkably prevented myocardial alterations after I/R, particularly interstitium edema and myocardial fiber disruption.

Presented in Fig. 5B are the representative ultrastructural images of myocardium in the four groups. As expected, the myocardium in sham group revealed a normal ultrastructure with regularly arranged myofibrils, well preserved sarcomeres and mitochondria. I/R challenge induced obvious injury in myocardium ultrastructure, including disrupted myofibrils and swelling mitochondria, which were protected by treatment with Rb1.

As a marker of myocardial damage, the level of cardiac troponin I (cTnI) in myocardium and blood plasma was determined using western blot and ELISA, respectively. The level of cTnI in myocardium decreased significantly in response to I/R challenge, as shown in Fig. 5C and D, as compared with sham group. In contrast, the level of cTnI in blood plasma was very low in sham group, but increased impressively after I/R, as shown in Fig. 5E. Of notice, I/R-elicited change in cTnI level in both myocardium and blood plasma was significantly protected by Rb1 treatment.

### Rb1 attenuates apoptosis in myocardium induced by I/R

**Figure 6:**
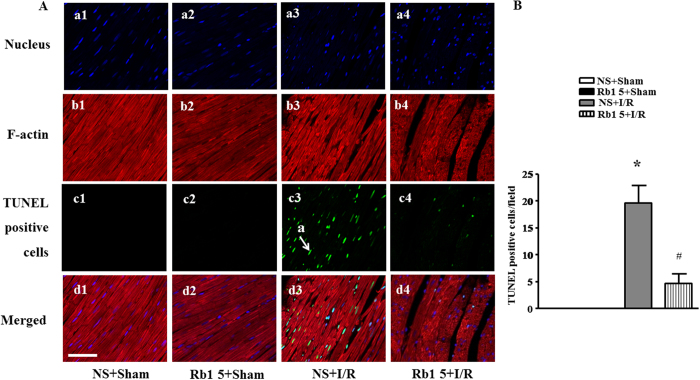
Effect of Rb1 on myocardial apoptosis and F-actin structure. (**A**) The representative photographs of rat myocardium with double staining for F-actin and TUNEL-positive apoptotic cells. Nuclei are stained with blue, F-actin red, and TUNEL-positive cells green (arrow). Bar = 50 μm, n = 3. (**B**) Quantification of apoptotic cardiomyocytes in various groups. Data are expressed as means ± S.E.M (n = 3). *p < 0.05 vs. sham group, ^#^p < 0.05 vs. I/R group.

Double staining of rhodamine phalloidine and transferase-mediated deoxyuridine triphosphate-biotin nick end labeling (TUNEL) was applied to assess the effect of Rb1 on the alteration in myocardium structure and apoptosis. As shown in Fig. 6A, a large number of TUNEL-positive cardiomyocytes (green) were observed at 30 min after reperfusion in I/R group, which was decreased in Rb1 treatment group. The statistical result of the percentage of TUNEL-positive cardiomyocytes in surrounding infarction areas validated the qualitative survey, as shown in Fig. 6B. Moreover, F-actin stained with rhodamine phalloidine (red) in I/R group showed severe rupture and abundant actin bundles. Apparently, the I/R-induced F-actin rearrangement was alleviated by Rb1.

**Figure 7:**
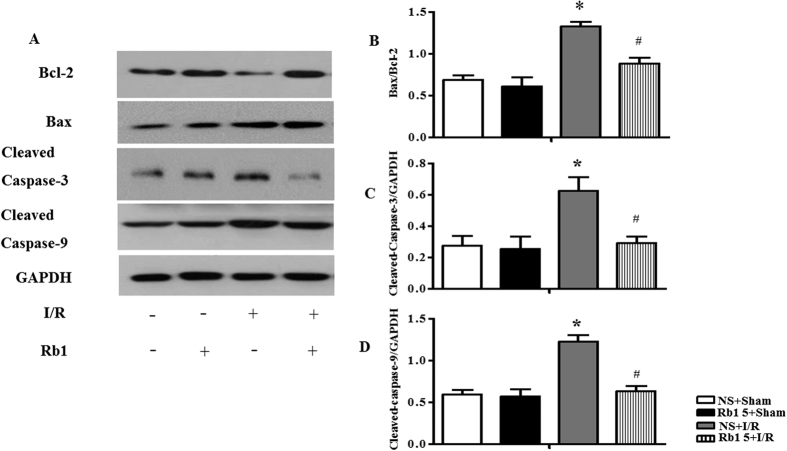
Effect of Rb1 on expression of apoptosis-related proteins after I/R. (**A**) Representative western blot bands of Bcl-2, Bax, cleaved caspase-3 and cleaved caspase-9 in cardiac tissue in different groups. GAPDH was used as a loading control. (**B**,**C**,**D** and **E**) The intensity of the western blot band was assessed by densitometry for Bax/Bcl-2, cleaved caspase-3 and cleaved caspase-9, respectively. The samples were derived from the same experiment and gels were processed in parallel. Full-length gels are shown in Supplementary Fig. S7 with indication of molecular size. Data are expressed as the means ± S.E.M (n = 4). *p < 0.05 vs. sham group, ^#^p < 0.05 vs. I/R group.

Apoptosis-related molecules were examined by Western blot assay, as shown in Fig. 7A with semi-quantitative analysis of western blot results presenting in Fig. 7B–E. B-cell Lymphoma-2 (Bcl-2), Bcl-2 Associated X protein (Bax), Caspase-3 and Caspase-9 are known as apoptosis-related proteins, with Bcl-2 being an anti-apoptosis factor, while Bax, Caspase-3 and Caspase-9 as pro-apoptotic molecules. The results of present study revealed that the ratio of Bax/Bcl-2, cleaved Caspase-3 and cleaved Caspase-9 expression significantly increased at 90 min after reperfusion, as compared to sham group, while these up-regulation was suppressed by treatment with Rb1. Taken together, these results suggested that treatment with Rb1 alleviated I/R-induced apoptosis.

### Rb1 targets RhoA and regulates the balance of RhoA signaling pathway during I/R

**Figure 8:**
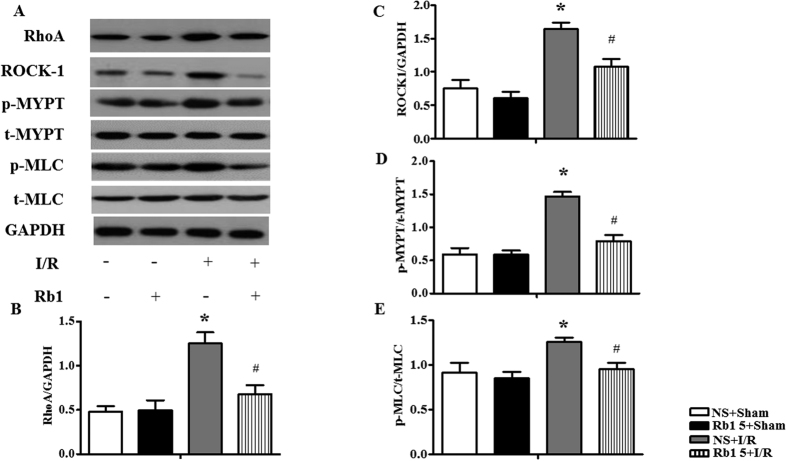
Effect of Rb1 on RhoA, ROCK-1, p-MYPT and p-MLC in rat myocardium subjected to I/R. (**A**) The representative Western blot bands of RhoA, ROCK-1, p-MYPT and p-MLC in myocardium in different groups. GAPDH was used as a loading control. (**B**,**C**,**D** and **E**) The semi-quantitative analysis of RhoA, ROCK-1, p-MYPT/t-MYPT and p-MLC/t-MLC respectively. The samples were derived from the same experiment and gels were processed in parallel. Full-length gels are shown in Supplementary Fig. S7 with indication of molecular size. Data are expressed as the means ± S.E.M (n = 4). *p < 0.05 vs. sham group, ^#^p < 0.05 vs. I/R group.

The expressions of RhoA and ROCK-1 were assessed by Western blot, and the results are shown in Fig. 8. Compared to sham group, I/R elevated significantly the expressions of RhoA (Fig. 8A and B) and ROCK-1 (Fig. 8A and C). Rb1 restrained all the alterations induced by I/R significantly.

As important effectors of ROCK1, the phosphorylation of MYPT-1 and MLC in various conditions was also examined. The expression of p-MYPT-1 and p-MLC increased significantly after I/R compared to sham group, as shown in Fig. 8A,D and E. Rb1 treatment significantly inhibited the phosphorylation of MYPT-1 and MLC evoked by I/R.

**Figure 9:**
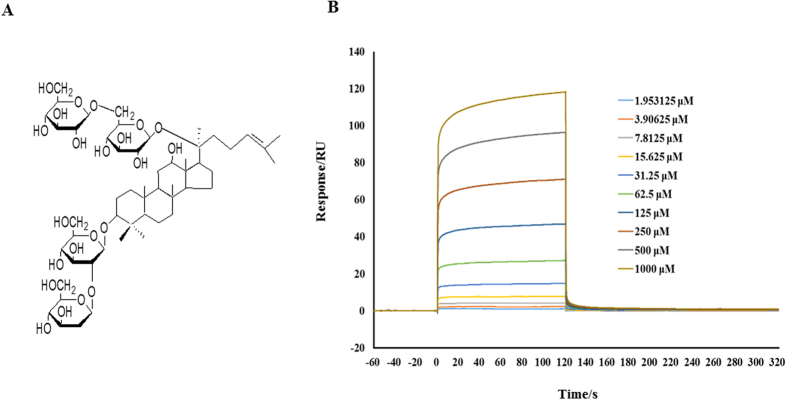
Rb1 interacts with RhoA. (**A**) The chemical structure of Rb1. (**B**) The affinity of Rb1 to RhoA tested by SPR. Shown are the representative sensorgrams obtained from the injections of Rb1 at concentrations of 1.953125, 3.90625, 7.8125, 15.625, 31.25, 62.5, 125, 250, 500 and 1000 μM, respectively (curves from bottom to top) using SPR.

The question was what was the target Rb1 acted at? Given the fact that RhoA and its downstream effectors have been reported to induce several energy metabolic changes, it is thus likely that Rb1 regulates RhoA/ROCK signaling pathway through binding and inactivating RhoA. To test this hypothesis, the binding capacity of Rb1 to RhoA was analyzed by surface plasmon resonance (SPR) assay. The results of SPR, as shown in Fig. 9B, indicated that Rb1 bound to RhoA in a dose-dependent manner. The equilibrium dissociation constant (K_D_) of Rb1 binding to RhoA was 2.835 * 10^−4^ (M).

### Rb1 restores ATP synthesis in myocardium during I/R

**Figure 10:**
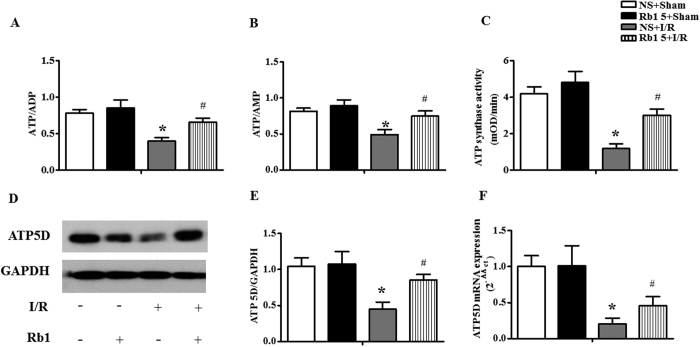
Effect of Rb1 on energy metabolism in rat myocardium subjected to I/R. (**A**) and (**B**) The effects of Rb1 on the ratio of ATP/ADP and ATP/AMP in myocardium in different groups. (**C**) Influence of pretreatment with Rb1 on the activity of ATP synthase in the myocardial tissue in various groups. (**D**) and (**E**) Western blot bands of ATP 5D and respective quantification in different groups. GAPDH was used as a loading control. Full-length gels are shown in Supplementary Fig. S10 with indication of molecular size. (**F**) Expression of ATP5D mRNA in myocardium of rats detected by Real-time PCR. All Data are expressed as the means ± S.E.M. n = 8 in (**A**,**B** and **C**); n = 4 in (**E** and **F**). *p < 0.05 vs. sham group, ^#^p < 0.05 vs. I/R group.

To assess the energy metabolism, the ratios ATP/ADP and ATP/AMP in cardiac tissue were determined in different conditions. As compared with NS + sham group, Rb1 treatment alone had no effect on either ATP/ADP or ATP/AMP (Fig. 10A and B). I/R challenge decreased ATP/ADP and ATP/AMP considerably, indicating a disorder in the balance of energy metabolism inclining toward ATP catabolism. Treatment with Rb1 significantly prevented both ATP/ADP and ATP/AMP from decreasing by I/R. Figure 10C shows the effect of Rb1 on the ATP synthase activity in myocardial tissue in various groups. The activity of ATP synthase diminished significantly after I/R, as compared to sham group, which was significantly protected by treatment with Rb1.

We then determined the protein and mRNA expression of ATP 5D, one subunit of ATP synthase, in cardiac tissue. As shown in Fig. 10D and E, ATP 5D protein expression reduced significantly after I/R, in comparison with sham group, which was prevented by treatment with Rb1. mRNA expression of ATP 5D followed a similar trend to the protein expression (Fig. 10F), indicating an enhanced transcription of ATP 5D by Rb1 treatment.

## Discussion

In this study, we found that Rb1 significantly inhibited I/R-induced rat myocardial injury, as demonstrated by the decrease of infarct size and cardiac apoptosis, together with improvement of myocardial morphology and function. In addition, Rb1 administration relieved myocardial energy disorders after I/R challenge, manifested by the increase of ATP 5D mRNA and protein expression, as well as ATP synthase activity. Furthermore, we provided evidence showing, for the first time, that Rb1 binds directly with RhoA, and inactivates RhoA/ROCK1 signaling pathway, thus interferes in its downstream targets. These findings suggest Rb1 as a potential alternative treatment for patients presenting with acute myocardial infarction, and that treatment with Rb1 may prevent reperfusion injury and improve PCI outcome.

Energy metabolism disorder and resultant ATP deficiency occurs in I/R injured myocardium, which is responsible for a diversity of insults, including cation pumps dysfunction and calcium overload, and excessive production of reactive oxygen species^14^. Restoration of the energy metabolism disorder in cardiomyocytes is thus believed to be potential strategies to attenuate cardiac I/R injury. We have reported that astragaloside IV and notoginsenoside R1, the two saponins derived from different sources, are able to prevent I/R-induced cardiac malfunction and maintain the integrity of myocardial structure through regulating energy metabolism^4^,^15^. In the present study we showed Rb1, another chemical belonging to the saponin family, protecting against cardiac I/R injury via regulation of energy metabolism as well, and gained insight into the target and signaling pathway implicated. Rb1 (chemical structure is shown in Fig. 9) has previously been reported exhibiting potential to protect against I/R injury via a range of mechanisms including anti-apoptosis^16^,^17^, anti-inflammation^18^,^19^, inhibiting oxidative stress^20^,^21^ and enhancing the expression of eNOS thus increasing the content of NO^22^. Some study also showed that Rb1 has an estrogen like effect and can activate estrogen-related intracellular pathways^23^. However, little is known about the effect of Rb1 on the energy metabolic disorders and changes in mitochondria induced by I/R challenge. In the present study, we observed a significant decrease in ATP content, as well as in ATP 5D expression and ATP synthase activity in myocardium after I/R, indicating presence of mitochondria dysfunction and ATP deficiency. Interestingly, Rb1 treatment protected all these I/R-induced energy metabolism disorders in myocardium, as well as myocardium impairment, which pointed to the potential of Rb1 to relieve the disordered energy metabolism by regulation of ATP5D expression and ATP synthase activity.

We then asked what was the target and signaling pathway that mediated Rb1 effect on energy metabolism? We speculated that it is RhoA that serves as the target for Rb1 action. RhoA is a small GTPase protein of Rho family and is primarily associated with cytoskeleton regulation, change in cell development, transcriptional control, among others^24^,^25^,^26^. The activation of the RhoA protein will lead to the activation of ROCK1, a kinase that belongs to PKA/PKG/PKC family of serine/threonine protein kinases. RhoA/ROCK activation was found previously to aggravate myocardial I/R injury by mediating deleterious role like myocardial apoptosis and inflammation^27^,^28^. The substrates of ROCK1 includes MYPT-1, the major effector of ROCK-mediated Ca^2+^ sensitization pathway of smooth muscle contraction^29^ which can be phosphorylated during I/R injury^30^, and MLC, which modulates smooth muscle cell contraction and plays a crucial role in tumor cell migration and metastasis^31^,^32^. More importantly, our previous study also demonstrated that RhoA/ROCK signaling pathway is one of the modulator of ATP5D expression and ATP production, and that NR1, a major effective ingredient of Panax notoginseng, was able to prevent I/R-induced energy metabolism disorder via inhibiting ROCK1^4^. In addition, a proteomic study by Cadete *et al*. showed that treatment with Y-27632, a ROCK inhibitor, results in increased levels of two different molecular fragments of ATP synthase during cardiac I/R injury, suggesting the involvement of ROCK in the impaired energy production^33^. The aforementioned evidence highlights a possibility that an agent that interferes in RhoA and its downstream signaling may affect energy metabolism thus influence myocardium structure and function. Supporting this speculation, the present study demonstrated that Rb1 prevented I/R-enhanced expression of RhoA and activation of its downstream signaling, including ROCK1, MYPT and MLC, concurrently attenuating energy metabolism disorder and myocardium impairment. A further support for this speculation comes from the result of SPR assay, thereby we showed for the first time that Rb1 binds to RhoA with high affinity, highly suggesting RhoA as the target for the action of Rb1. Nevertheless, involvement of other signaling pathways in Rb1 action needs to be precluded by further study.

As a limitation of this study, we failed to detect any effect of Rb1 on dp/dtmax during I/R, the reason for which is at present unknown and needs further study to elucidate.

In summary, Rb1 was able to target and inactivate RhoA and downstream signaling, which likely mediated the protective role of Rb1 in I/R-induced energy metabolism disorder and cardiomyocyte apoptosis, providing an explanation for the restoration of heart structure and function by Rb1 after I/R. These results suggested Rb1 as a potential regime to protect cardiac I/R injury.

## Materials and Methods

### Animals

Male Sprague-Dawley (SD) rats weighing 230–270 g were obtained from the Animal Center of Peking University (certificate no. SCXK (Jing) 2006–0008). The rats were raised in cages at temperature 22 ± 2 °C and relative humidity 40 ± 5% under a 12-hour light/dark cycle, with standard diet and water *ad libitum*. The rats were fasted for 12 hours before experiment while allowing to reach water freely. The investigations complied with the Guide of Peking University Animal Research Committee. All experimental procedures were approved by Peking University Biomedical Ethics Committee Experimental Animal Ethics Branch (LA2010-001).

### Regents

Rb1 was purchased from Feng Shan Jian Medicine Research Co. Ltd. (Kunming, Yunnan, China), and dissolved in saline to make a solution of concentration of 1.25 mg/ml before experiment. Urethane was from Beijing Chemical Agent Ltd (Beijing, China).

### Cardiac I/R model and experiment protocols

Animals were anesthetized with urethane (1.25 g/kg, i.m), and placed in a dorsal position. A tracheal cannula was inserted via mouth with one end connected to an animal breathing apparatus (ALC-V8; Shanghai Alcott Biotech Co., Shanghai, China), which was set at the breathing ratio 1:1, the frequency 75 times/min, and tidal volume 12 ml/kg. A thoracotomy was carried out to expose the heart, and the left anterior descending coronary artery was ligated with a 5/0 silk. The suture silk was released after 30 min, allowing reperfusion for 90 min. The animals in NS + sham and Rb1 + sham groups underwent the same procedure except for ligation of suture silk. Thirty min before ischemia, the animals in Rb1 + I/R groups received Rb1 by continuous infusion through femoral vein at 2.5, 5 or 7.5 mg/kg/h. The animals in NS + sham group and NS + I/R group received saline in the same way at the same speed. The number of animals enrolled in each group for determination of each parameter is detailed in Table 1.

### MBF

MBF was determined at indicated time points using a Laser-Doppler Perfusion Imager (PeriScan PIM3 System; Perimed, Stockholm, Sweden). Left thoracotomy was performed to expose the heart. A computer-controlled optical scanner directed a low-powered He-Ne laser beam over the exposed heart with the scanner head positioning in parallel to the surface of heart at a distance of 18 cm. The laser beam illuminated the tissue to a depth of 0.5 mm. A color-coded image was displayed on a video monitor, and evaluated with the software LDPIwin 3.1 (PeriScan PIM3 System; Perimed, Stockholm, Sweden). The magnitude of MBF was graded by different colors, with blue to red denoting low to high. Results were presented as percent of the baseline MBF^13^.

### Heart function test

Heart function was tested by a bio-function experiment system BL-420F (Chengdu Taimen Technology Ltd, Chengdu, Sichuan, China), which was connected to a cannulation inserted into left ventricle (LV) through right carotid artery. HR, LVSP, LVDP, LVEDP, left + dp/dtmax, −dp/dtmax were evaluated at the indicated time points^13^.

### Myocardial infarct size

LADCA was ligated 90 min after reperfusion, and 2 ml of 0.35% Evans blue (Sigma, St. Louis, MO, USA) was infused via femoral vein. Hearts were removed and cut into five sections (1 mm thick), parallel to the atrioventricular groove from the apex cordis to the ligation site. Sections were incubated in a 0.375% solution of TTC (Sigma, St. Louis, MO, USA) at 37 °C for 15 min, and then photographed using a stereomicroscope connected with Digital Sight (DS-5M-U; Nikon, Nanjing, Jiangsu, China). In so treated sections, the degree of myocardial tissue injury was discriminated by different colors with infarction zone (IA) being stained white, area at risk (AAR) pink, and non-infarction zone blue. The myocardial IA, AAR and LV in each section was determined by Image-Pro Plus 6.0 (Media Cybernetic, Bethesda, MD, USA), and the ratios AAR/LV (%) and infarct area/AAR (%) were calculated. The values from five sections were averaged and used to score the degree of myocardial infarction^13^.

### RBC velocity in coronary venules

An upright microscope (BX51WI, Olympus, Tokyo, Japan) connected with a high-speed video camera (APX, Photon Fastcam-ultimate, Tokyo, Japan) using epi-illumination was applied to assess the hemodynamics of coronary venules (25–40 μm in diameter). During observation, saline of 37 °C was continuously superfused over the heart to keep it warm and moist. A monitor (20PF5120, Philips, Amsterdam, Netherlands) and a DVD videocassette recorder (DVR-560H, Philips, Amsterdam, Netherlands) were used to acquire and display the images. The RBC motion in venules was recorded for 4 s at a rate of 500 frames/s, and the stored images were replayed at a rate of 25 frames/s. The RBC velocity was determined using Image-Pro Plus 5.0 software (Media Cybernetic, Bethesda, MD, USA) at the indicated time points. Results were presented as percentages of the baseline^34^.

### Double staining of rhodamine phalloidine and TUNEL

Rat heart was perfused with saline followed by removal and fixation in 4% paraformaldehyde solution for 48 hours. To reveal F-actin and TUNEL-positive apoptotic cells, heart paraffin section (5 μm) was prepared and subjected to double staining with rhodamine phalloidine (R415; Invitrogen, Carlsbad, CA, USA) and cell death detection kit (Roche, Basel, Switzerland), according to the manufacture’s instruction. The nuclei were labeled with Hoechest33342. Five fields were selected from the surrounding infarction areas of the LV for each section at 40x magnification of objective, and observed with a Laser Scanning Confocal Microscope (TCS SP5; Leica, Mannheim, Germany)^13^.

### Ultrastructure examination

Rat hearts were perfused for 40 min with 4% paraformaldehyde and 2% glutaraldehyde (TedPella, Redding, CA, USA) in 0.1 mol/L phosphate buffer at a speed of 3 ml/min, after which the hearts were removed. Myocardial tissue was collected at one third above the apex cordis from the surrounding infarct region of left ventricle and cut into 1 mm^3^ blocks. The tissue blocks were fixed overnight at 4 °C with 3% glutaraldehyde, washed 3 times with 0.1 mol/L phosphate-buffered solution, and then post-fixed with 1% osmium tetraoxide for 2 h. The ultrathin sections were prepared as routine, observed and photographed with a transmission electron microscope (JEM 1230, JEOL, Tokyo, Japan)^13^.

### Histological evaluation of myocardial tissues

Hearts were removed from the middle one third between the apex and the ligation point at 90 min after reperfusion, fixed in 10% formalin, and then prepared for paraffin sectioning. The paraffin sections (5 μm) were stained with HE and observed and photographed by a microscope equipped with a digital camera (BX512DP70, Olympus)^13^.

### SPR

SPR, a technique able to detect biomolecular association and dissociation without labeling, is highly sensitive to monitor molecular interactions in real time. A Carboxymethylated 5 (CM5) sensor chip (GE Healthcare Life Sciences, London, England) was docked into a Biacore T200 (Biacore, GE Healthcare, Sweden) and prepared as previously reported^35^. Human RhoA full-length protein (Abcam, Cambridge, MA, USA) was immobilized on a CM5 sensor chip by injecting 40 μl of RhoA (1 μg/μl in 10 mM sodium acetate, pH 4.5) at a rate of 5 μl/min. Rb1 was prepared as a 2000 μM solution in a running buffer before the experiment and diluted twofold by running buffer to 1000 μM, 500 μM, 250 μM, 125 μM, 62.5 μM, 31.25 μM, 15.625 μM, 7.8125 μM, 3.90625 μM, 1.953125 μM before injection. Analytes were injected at 30 μl/min over RhoA and a control sensor chip. A quantity of 90 μl of each Rb1 solution was injected and 300 s was set as the dissociation time. No regeneration solution was required because all Rb1 solutions were removed from the surface. Samples were injected from low to high concentration to eliminate artifacts in the data from adsorption carryover on the instrument flow. Equilibrium dissociation constant (K_D_) was calculated by fitting a 1:1 Langmuir model using Biacore T200 evaluation software v2.0 (Biacore, GE Healthcare, Sweden)^36^.

### Protein extraction

Rat hearts were removed after animals were perfused with saline under anesthesia. The LV tissue sample was taken at 2 mm under ligature, frozen in liquid nitrogen, and stored at −80 °C before use. The sample was thawed and centrifuged at 20,000 *g* to fracture the membrane. Total tissue protein was extracted using a total protein extraction kit (Applygen Technologies, Beijing, China), according to manufacturer’s instruction.

### Western blot

The concentration of total protein of each sample was determined twice with a BCA protein assay kit (Applygen Technologies), according to the manufacture’s instruction, taking the average as the concentration. The protein was mixed with 2x electrophoresis sample buffer, separated on 8% or 10% SDS-PAGE, and transferred to polyvinylidene difluoride membrane. The membrane was blocked with 3% skimmed milk powder, rinsing with TBS-Tween for three times, 5 min each, and then cut and incubated overnight at 4 °C with antibodies, respectively, against glyceraldehyde-3-phosphatedehydrogenase (GAPDH), p-MLC, MLC, Bcl-2, Bax, cleaved-Caspase-3, cleaved-Caspase-9, cTnI (1:1000, Cell Signaling Technology, Berley, MA, USA), RhoA, ROCK1, MYPT, p-MYPT (1:1000, Abcam, Cambrige, MA, USA), ATP5D (1:200, Santa Cruz, California, USA). Following rinsing three times, 5 min each, the membrane was incubated with secondary antibody for 1 hour at room temperature, rinsed with TBS-Tween three times, 10 min each. The Quantification of target protein was carried out by scanning densitometry in the X-film using a bio-image analysis system (Image-Pro plus 6.0; Media Cybernetic, Bethesda, MD, USA)^13^.

### Content of ATP, ADP, and AMP in myocardium

Content of ATP, ADP, AMP in myocardium was determined with ELISA using a microplate reader (MULTISKAN MK3, Thermo, San Jose, CA, USA) according to the manufacturer’s instructions^13^.

### cTnI content in serum

Blood was collected at 90 min after reperfusion and serum prepared using heparin as an anticoagulant. The samples were centrifuged for 15 min at 1000 *g,* the supernatant was collected for determination of the cTnI content using rat cTnI ELISA Kit by microplate reader (MULTISKAN MK3; Thermo, San Jose, CA, USA)^15^.

### Real-time quantitative PCR test of ATP 5D

To detect mRNA level of ATP 5D, real-time quantitative PCR was performed according to the manufacturer’s instruction. For this purpose, 30 mg of myocardial tissue from the surrounding of infarct area of LV was sampled and stored at −80 °C. RNA was extracted using RNeasy Fibrous Tissue Mini Kit (Qiagen, Hilden, Germany), according to the manufacturer’s protocol and applied for reverse transcription using a RevertAid First Strand cDNA Synthesis Kit (Fermentas Lifesciences, Vilnius, Lithuania) to generate the first strand cDNA mix. Real-time PCR was undertaken using the ABI PRISM sequence detection system 7500 (Perkin-Elmer Applied Biosystems, CA, USA). The primer sequences (all Rattus) applied were as follows: ATP 5D—forward, 5′-CACTGTG AATGCG./GACTCCT-3′; reverse, 5′-GGATTTGGATCTCAG CCCGT-3′; GAPDH—forward, 5′-AGTTCAACGGCACAG TCAAG-3′; reverse, 5′-TACTCAGCACCAGCATCACC-3′. The PCR reaction mixture (25 μl) contained 2x Maxima SYBR Green/ROX qPCR Master Mix, reverse transcription product cDNA, forward and reverse primers, nuclease-free water. The reactions took place in a 96-well plate at 50 °C for 2 min, 95 °C for 10 min, followed by 40 cycles of 95 °C for 15 seconds, 58 °C for 1 min and plate read. Each experiment was conducted in triplicate^15^.

### ATP synthase activity

Total protein concentration of myocardium was detected with BCA protein assay kit (Applygen, Beijing, China) and adjusted to a concentration of 5.5 mg/ml. The sample was added with detergent at a ratio 1/10 (v/v), mixed and then incubated on ice for 30 min. Following centrifugation at 12,000 *g* for 20 min, the supernatant was collected. ATP synthase activity was determined using an ATP Synthase Enzyme Activity Microplate Assay Kit (Abcam, Cambridge, UK), with setting the plate in the MULTISKAN MK3 enzyme micro-plate reader (Thermo Fisher Scientific Inc., Illinois, USA). The absorbance of each well was measured at 340 nm, 30 °C, for 60 min using a kinetic program. The activity was expressed as the change in absorbance at 340 nm (mOD/min)^4^.

### Statistical analysis

All data were expressed as mean ± S.E.M. Statistical analysis was performed using one-way ANOVA followed by Newman-Keuls test or using two-way ANOVA followed by Bonferroni for multiple comparisons (MBF and cardiac function). Data were analyzed using GraphPad Prism 5 software (GraphPad software Inc., USA). A *p* value less than 0.05 was considered to be statistically significant.

## Additional Information

**How to cite this article**: Cui, Y.-C. *et al*. Ginsenoside Rb1 protects against ischemia/reperfusion-induced myocardial injury via energy metabolism regulation mediated by RhoA signaling pathway. *Sci. Rep.* **7**, 44579; doi: 10.1038/srep44579 (2017).

**Publisher's note:** Springer Nature remains neutral with regard to jurisdictional claims in published maps and institutional affiliations.

## Supplementary Material

Supplementary Information

## Figures and Tables

**Table 1:** Animal number in each group for different experiments.

	NS + sham	Rb1 5 + sham	NS + I/R	Rb1 2.5 + I/R	Rb15 + I/R	Rb17.5 + I/R	Total
*Myocardial blood flowand myocardial infarct size	6	6	6	6	6	6	36
HE staining	3	3	3		3		12
*Hemodynamics and ELISA	8	8	8		8		32
RBC velocity	6	6	6		6		24
F-actin-TUNEL double staining	3	3	3		3		12
Ultra-structure examination	3	3	3		3		12
Western blot assay and PCR	4	4	4		4		16
Total	33	33	33	6	33	6	144

*The two experiments were undertaken using the same set of animals. NS + sham: saline plus sham group; Rb1 5 + sham: Rb1 5 mg/kg/h plus sham group; NS + I/R: saline plus I/R group; Rb1 2.5 + I/R: treatment with Rb1 at 2.5 mg/kg/h plus I/R group; Rb1 5 + I/R: treatment with Rb1 at 5 mg/kg/h plus I/R group; Rb1 7.5 + I/R: treatment with Rb1 at 7.5 mg/kg/h plus I/R group.
