# Supplementary material for: Ginsenoside Rb1 protects against ischemia/reperfusion-induced myocardial injury via energy metabolism regulation mediated by RhoA signaling pathway
Source: Sci Rep. 2017 Mar 22;7:44579. doi: 10.1038/srep44579 (PMC5361119; doi:10.1038/srep44579)
Supplement: Supplementary Information [file srep44579-s1.doc]

**Ginsenoside Rb1 protects against ischemia/reperfusion-induced myocardial injury via energy metabolism regulation mediated by RhoA signaling pathway**

Yuan-Chen Cui^1,2,3,4,5^, Chun-Shui Pan^2,3,4,5^, Li Yan^2,3,4,5^, Lin Li^6^, Bai-He Hu^2,3,4,5^, Xin Chang^2,3,4,5^, Yu-Ying Liu^2,3,4,5^, Jing-Yu Fan^2,5^, Kai Sun^2,3,4,5^，Quan-Li^2,3,4,5^ and Jing-Yan Han^1,2,3,4,5^

^1^ Department of Integration of Chinese and Western Medicine, School of Basic Medical Sciences, Peking University, Beijing 100191, China

^2^ Tasly Microcirculation Research Center, Peking University Health Science Center, Beijing 100191, China

^3^ Key Laboratory of Microcirculation, State Administration of Traditional Chinese Medicine of the People's Republic of China, Beijing 100191, China

^4^ Key Laboratory of Stasis and Phlegm, State Administration of Traditional Chinese Medicine of the People's Republic of China, Beijing 100191, China

^5^ Beijing Laboratory of Integrative Microangiopathy, Beijing 100191, China

^6^ Department of Cardiology, Beijing China-Japan Friendship Hospital, Beijing 100029, China

**Supplementary Materials: Supplementary Figures S5, S7, S8 and S10**

**Figure S5.**

**
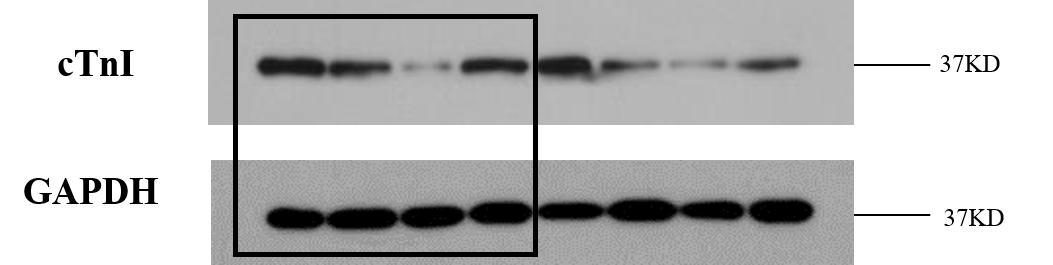
**

**Figure S5.** The full-length representative western blotting bands of cTnI and GAPDH in different groups as shown with indication of molecular size. The bands of cTnI and GAPDH were cropped from different gels because of its overlapping molecular size with GAPDH. The samples derived from the same experiment and gels were processed in parallel. Selected fractions of bands used in the main text were framed as shown in the figure.

**Figure S7.**


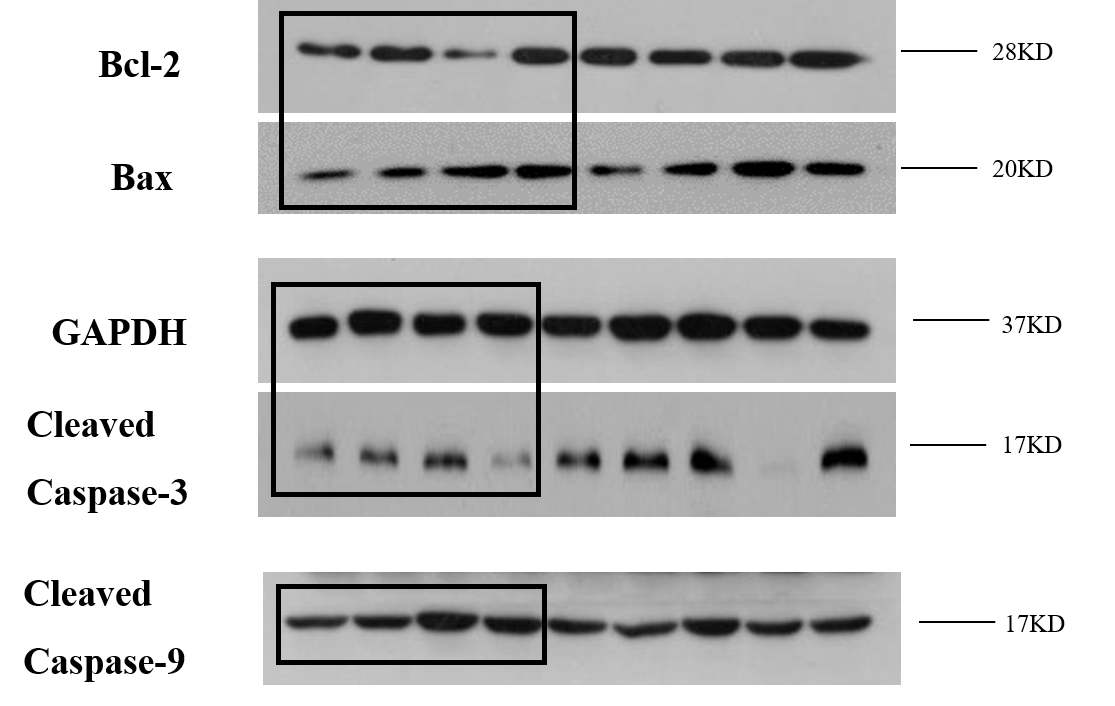


**Figure S7.** The full-length representative western blotting bands of Bcl-2，Bax，Caspase-3，Caspase-9 and GAPDH in different groups as shown with indication of molecular size. The bands of Bcl-2 and Bax were cropped from one gel, and the bands of Caspase-3 and GAPDH were cropped from one gel. The bands of Caspase-9 were cropped from another gel because of its overlapping molecular size with GAPDH. The samples derived from the same experiment and gels were processed in parallel. Selected fractions of bands used in the main text were framed as shown in the figure.

**Figure S8.**


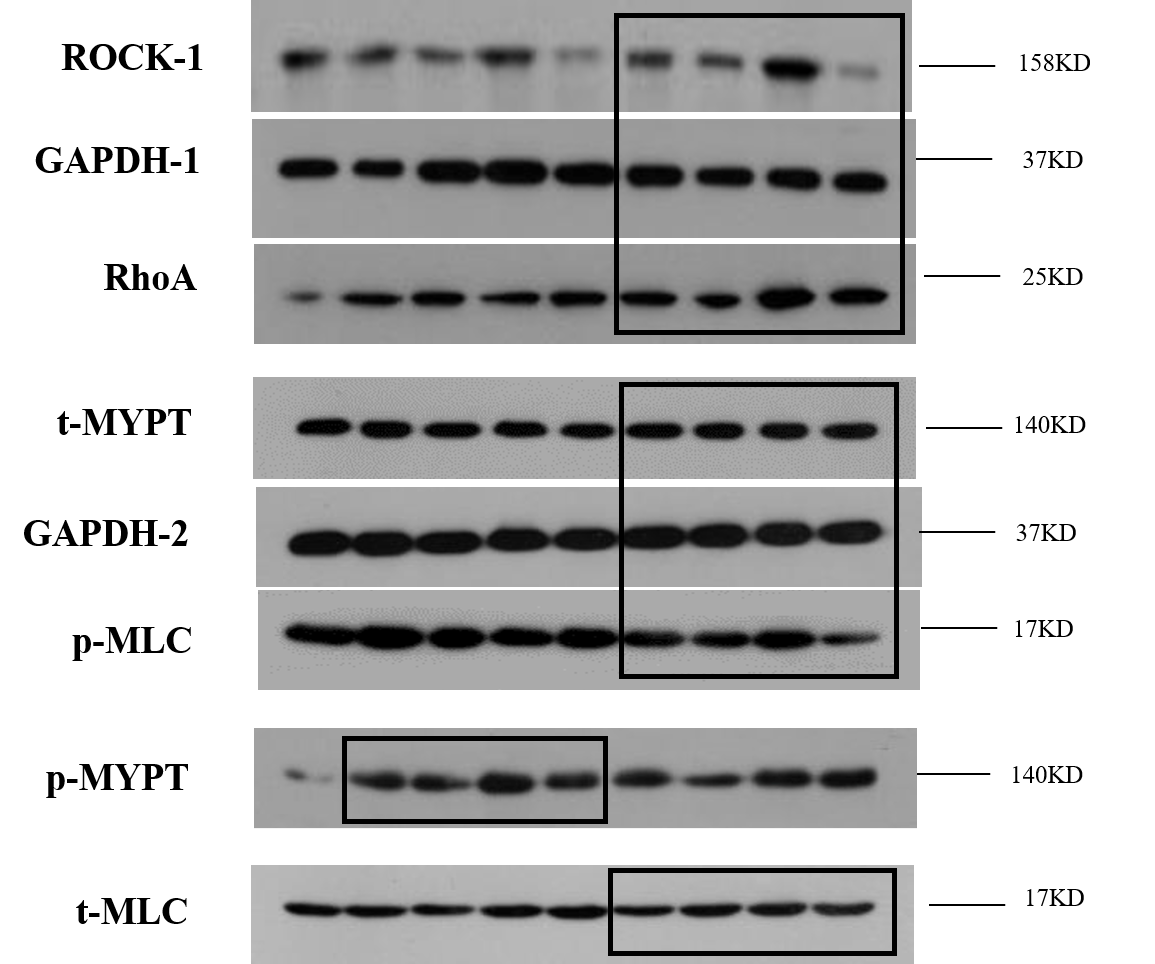


**Figure S8.** The full-length representative western blotting bands of RhoA，ROCK-1， p-MLC，t-MLC，p-MYPT，t-MYPT and GAPDH in different groups, as shown with indication of molecular size. The bands of RhoA, ROCK-1 and GAPDH-1 were cropped from one gel, and the bands of p-MLC, t-MYPT and GAPDH-2 were cropped from one gel, and the bands of p-MYPT and t-MLC were cropped from two other separated gels, respectively. The samples derived from the same experiment and gels were processed in parallel. Bands of total proteins and their phosphorylated forms had to be derived from different gels because of their overlapping molecular sizes. Selected fractions of bands used in the main text were framed as shown in the figure.

**Figure S10.**


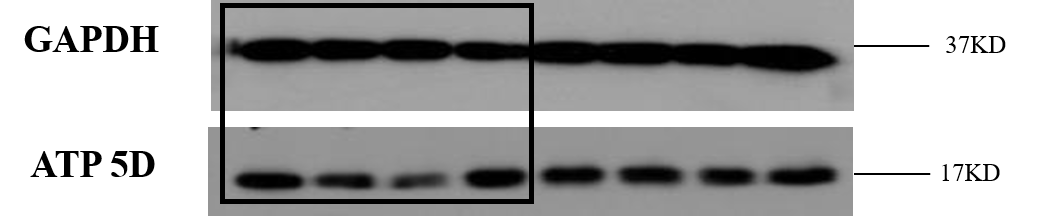


**Figure S10.** The full-length representative western blotting bands of ATP 5D and GAPDH in different groups, as shown with indication of molecular size. The bands of ATP 5D and GAPDH were cropped from one gel. Selected fractions of bands used in the main text were framed as shown in the figure.
